# NeuroSift for Task-Aware Quality Assurance of Multimedia Data in Remote Parkinson Disease Assessment: Machine Learning Model Development and Validation Study

**DOI:** 10.2196/91756

**Published:** 2026-08-18

**Authors:** Md Saiful Islam, Sooyong Park, Evelyn Xiaoxiao Ma, Tariq Adnan, Ehsan Hoque

**Affiliations:** 1 Department of Computer Science University of Rochester Rochester, NY United States

**Keywords:** movement disorders, remote assessment, machine learning, video analysis, audio analysis, data quality, telemedicine, digital health, neurological screening

## Abstract

**Background:**

Automated multimedia analysis of remotely recorded tasks offers a scalable approach to screening and remote monitoring of movement disorders such as Parkinson disease (PD). However, unsupervised recordings often suffer from quality issues that compromise model reliability. General multimedia quality checks may not detect task-specific failures, such as poor hand visibility during finger-tapping, inadequate facial framing during smile tasks, or background noise during speech tasks.

**Objective:**

This study aimed to develop and evaluate NeuroSift, a task-aware, interpretable machine learning framework for assessing recording quality and task compliance in home-recorded multimedia data collected for remote PD assessment.

**Methods:**

We analyzed 2516 home-recorded audio and video segments from 3 tasks: finger-tapping, facial expression (smile), and speech (pangram utterance). Three experts rated recordings as poor, borderline, or good quality. Task-specific annotation guidelines were developed through iterative review and discussion. We extracted interpretable features aligned with observable quality and compliance criteria and trained task-specific quality classification models. Interrater reliability was evaluated before and after guideline implementation using quadratic weighted Cohen κ (QWK), pairwise agreement, complete agreement, and intraclass correlation. Model performance was evaluated on a held-out test set (labeled using expert consensus) using accuracy, QWK, and ordinal classification accuracy (OCA, an accuracy metric that accounts for the ordered relationship among poor, borderline, and good labels). Feature importance was examined using Shapley additive explanations (SHAP), a method for estimating how individual features contribute to model predictions.

**Results:**

The task-specific guidelines significantly improved interrater reliability across all tasks (*P*<.001)—QWK increased from 0.46 to 0.89 for finger-tapping, from 0.61 to 0.84 for smile, and from 0.64 to 0.90 for speech. For 3-class quality classification, the best-performing models achieved QWK values of 0.71 for finger-tapping, 0.56 for smile, and 0.72 for speech. OCA was 82.1% for finger-tapping, 76.9% for smile, and 89.9% for speech. Most errors were adjacent-class errors, such as classifying poor recordings as borderline, whereas severe errors, such as classifying good recordings as poor, were rare. SHAP analyses identified task-specific sources of quality degradation that aligned with the annotation guidelines.

**Conclusions:**

NeuroSift was evaluated as a task-aware quality-classification framework for identifying low-quality or noncompliant multimedia data prior to downstream PD assessment. By combining structured quality guidelines, interpretable features, and explainable machine learning models, NeuroSift supports more transparent and user-correctable remote data collection. Although this study did not test downstream clinical impact, the framework offers a practical approach for quality-aware workflows that rely on user-recorded audio or video. This approach may extend beyond PD assessment to other remote settings, including telehealth, rehabilitation, and digital recruitment. Future studies should evaluate NeuroSift on independent datasets and examine its effects on downstream model performance, fairness, user experience, and workflow integration.

## Introduction

### Need for Automated Quality Assurance

Imagine an individual at home, trying to complete a simple finger-tapping or speech task for an AI-based health assessment. The setup looks straightforward, but the camera angle cuts off their hand, the lighting obscures their face, or the microphone captures more background noise than their voice. For a clinician, these errors are obvious. For an algorithm, they are harder. The system cannot easily distinguish between actual motor symptoms and poor video quality. Without safeguards, the very recordings meant to extend access can instead mislead or fail altogether.

Neurological movement disorders such as essential tremor, Huntington disease, and Parkinson disease (PD) affect tens of millions worldwide [1]. The number of people living with PD alone doubled from about 3 million in 1990 to over 6 million in 2015 and is projected to surpass 12 million by 2040 [2]. Yet, access to neurological care remains scarce: in many countries, there is less than 1 neurologist per 100,000 people [3,4], and even in well-resourced regions, rural and low-income communities face hours-long travel for appointments [5]. Older adults, the most affected group, are often least able to make these trips, leaving many undiagnosed or untreated [6,7]. Remote assessment offers a promising alternative, enabling frequent and accessible evaluations without the burden of in-person visits [8].

Compared to wearable sensors, audio/video (A/V) recordings may have clear advantages. Sensors require shipping, setup, and device literacy, which can be daunting for older users [9]. In contrast, A/V can be captured using everyday devices like smartphones or webcams, preserving the clinically familiar cues of movement and speech [10]. Recent studies show that automated A/V analysis can support screening and progression tracking for finger-tapping [9,11], facial expression [12,13], and speech tasks [14,15]. While these assessments provide a scalable means for remote screening [16], they cannot address the shortage of neurologists if clinicians must still supervise recordings. Unsupervised home-recorded multimedia data, though promising for detecting disorders such as PD [17] and ataxia [18], introduces new challenges. Older adults may struggle to follow instructions [19], and uncontrolled environments often result in poor lighting, occlusion, or distracting background noise. In clinics, staff can intervene to correct these issues, but at home, no such safeguard exists. As a result, predictive models trained on low-quality data risk basing their decisions on noise rather than genuine symptoms, undermining both trust and clinical utility. Ensuring automated quality checks is therefore essential to safeguard patient safety and preserve the reliability of machine learning systems.

Movement disorder screening is task-based, with each task targeting specific clinical symptoms [20]. While general A/V quality checks (eg, background clutter or lighting) are helpful, they are insufficient for assessing task compliance. For example, finger-tapping tasks [21,22] require clear visibility of the thumb and index finger; facial expression tasks [12] require unobstructed frontal views; speech tasks [23] require clean audio without noise. Existing quality checks [24,25] typically address generic capture issues, leaving compliance to manual review [26]. A prior study [27] has also emphasized the need for clearer instructions, camera framing assistance, and real-time quality feedback, suggesting that built-in guidance and automated checks could enhance both task compliance and user engagement.

**Figure 1:**
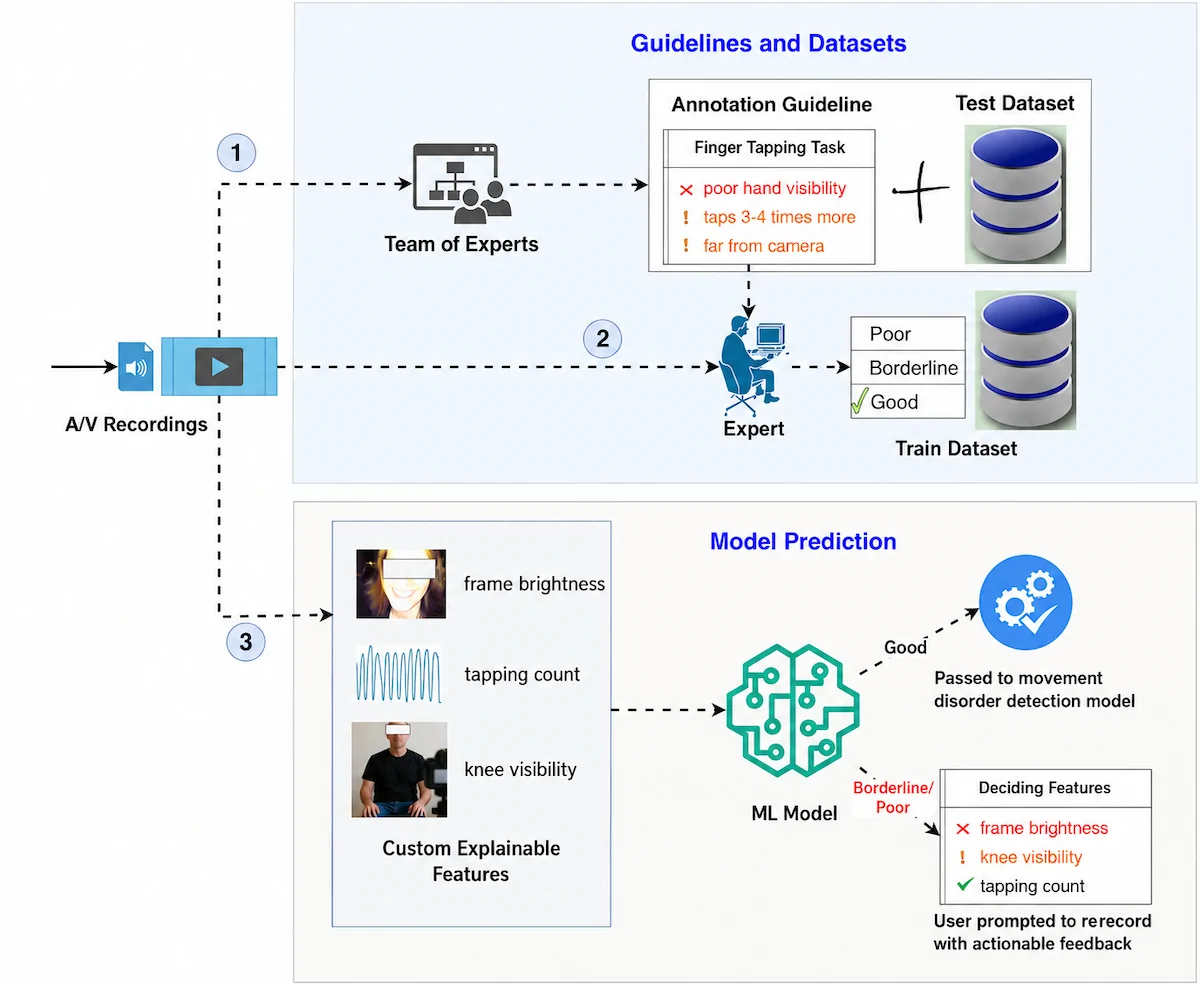
Overview of the proposed multimedia data quality assurance framework. (1) Three experts first independently rated audio/video recordings of finger-tapping, facial expression, and speech tasks. Through collaborative discussion on 100+ samples per task, they established task-specific annotation guidelines and created a consensus-labeled test set. (2) Using these guidelines, experts independently annotated additional recordings to build the training dataset. (3) We then engineered explainable features aligned with the guidelines and trained machine learning models to predict data quality. High-quality data can be passed to the movement disorder detection model, while low-quality data may trigger Shapley additive explanations (SHAP)–based feedback highlighting key issues and prompting users to rerecord. A/V: audio/video; ML: machine learning.

We address this gap with *NeuroSift* (see Figure 1 for an overview), a task-aware quality assurance framework for 3 remotely collected tasks used in PD assessment: finger-tapping, facial expression (smile), and speech (pangram utterance) tests. Although our dataset is collected primarily in the context of PD assessment [21], these tasks capture functional domains that are also relevant across Huntington disease [28], cerebellar ataxia [29], amyotrophic lateral sclerosis (ALS) [30], dystonia [31], and other neurodegenerative conditions. Each of these tasks introduces unique quality and compliance considerations: hands may drift out of frame during tapping, facial expressions may be obscured by poor lighting, and speech may be masked by background noise. To address these challenges, we curate a dataset of 2516 home-recorded audio or video segments spanning these tasks, annotate each segment with expert-provided quality labels, and develop task-specific quality guidelines. These guidelines help raters to assess observable quality issues objectively and significantly improve interrater agreement (at least a 0.23 gain in weighted Cohen κ score across all 3 tasks; *P*<.001) while offering practical checklists for both clinicians and study designers.

Building on these guidelines, we engineer interpretable computational features—13 for finger-tapping, 16 for facial expression, and 6 for speech—directly tied to observable aspects of quality and compliance. Using these features, we train machine learning models that achieve 83%, 77%, and 90% ordinal accuracy in predicting 3-class quality labels (poor, borderline, and good) across the finger-tapping, facial expression, and speech tasks, respectively. Beyond performance, the feature-based models support explainability. Using Shapley additive explanations (SHAP [32]), a model-interpretation method that estimates how much each input feature contributes to a prediction, we can identify the key factors (eg, hand visibility, facial illumination, and background noise) that drive model prediction. As a result, instead of a “black-box” rejection, users can potentially receive actionable feedback: why the recording failed and how to fix it.

The aim of this study is to develop and evaluate *NeuroSift* as a task-aware, interpretable quality-assurance framework for user-recorded multimedia data in remote movement disorder assessment. Specifically, we ask: (1) Can structured, task-specific annotation guidelines improve expert agreement when rating recording quality and task compliance? (2) Can interpretable, task-specific features support automated classification of recording quality across finger-tapping, facial expression, and speech tasks? (3) Can model explanations identify actionable sources of recording failure that may guide users toward successful rerecording? By addressing these questions, this study evaluates whether automated quality assurance can serve as a practical safeguard for scalable, reliable, and transparent remote neurological assessment, using PD as a specific use case.

### Background and Related Works

#### Overview

In this section, we outline how movement disorders are assessed, moving from traditional clinician ratings to sensor-based approaches and, more recently, A/V recordings. We focus on recurring challenges of quality, compliance, and safety, and highlight the importance of core tasks such as finger-tapping, facial expressions, and speech. We then review current approaches to automated A/V quality checks, pointing to key gaps that set the stage for our work.

#### Assessment of Movement Disorders

##### Traditional Clinical Assessments

Clinical evaluation of movement disorders has traditionally relied on scales such as the MDS-UPDRS (Movement Disorder Society–Sponsored Revision of the Unified Parkinson’s Disease Rating Scale) [20], Hoehn and Yahr [33] staging for PD, and analogous instruments for Huntington disease, ataxias, dystonia, and essential tremor [34-38]. These tools remain central to diagnosis and tracking, but face critical challenges: limited temporal sampling due to brief clinic visits [39] and high resource burden of administration [40]. Telemedicine has extended these scales into remote contexts, with video-based scoring of PD and ataxia showing promising reliability for many visual tasks [18,41]. However, due to the shortage of expert neurologists, clinician-led review remains a bottleneck and does not scale to continuous or population-level monitoring of movement disorders.

##### Wearable and Smartphone Sensing

Wearable and smartphone-based sensors offer objective, continuous tracking of motor symptoms. Devices such as the Parkinson KinetiGraph can quantify bradykinesia and dyskinesia, supporting management in routine PD care [24]. Similarly, inertial sensors capture gait variability that correlates with ataxia severity (SARA, Scale for the Assessment and Rating of Ataxia) [42]. Reviews highlight their validity across multiple conditions [43], and systematic studies also point to the promise of wearables for remotely capturing motor symptoms in Huntington disease. However, standardization remains limited [44]. More broadly, inertial measurement unit (IMU)–based devices provide excellent reliability for spatiotemporal gait parameters, making them well-suited for longitudinal monitoring. While sensors excel at precision and continuous tracking, their reliance on specialized hardware, calibration, and patient adherence limits their scalability.

##### Contactless RF (Wi-Fi) Sensing

Beyond wearable devices, recent studies have explored passive sensing using radio frequency (RF) reflections from Wi-Fi signals to capture physiological and motor biomarkers. These systems enable unobtrusive, continuous monitoring in home environments without requiring worn sensors or active user engagement. For instance, in-home Wi-Fi sensing has been shown to continuously track gait in individuals with PD, collecting over 200,000 gait measurements across 1 year and correlating strongly with disease severity, progression, and medication response [45]. Respiration patterns can also be reliably measured using commodity Wi-Fi devices under natural body orientations [46]. Subsequent work has extended these methods to robust vital-sign monitoring during free movement in daily life [47]. Despite these advances, RF-based systems remain sensitive to multipath interference, occlusion, and environmental variability, and often require careful calibration, domain adaptation, and motion-artifact suppression to maintain robustness across users and home layouts.

##### Multimedia-Based Assessments

Multimedia, especially audio and video (A/V), offers a scalable alternative, leveraging ubiquitous cameras and microphones to capture multimodal signals indicative of the disease. Advances in 2D/3D pose estimation show strong alignment between automated video features and clinician ratings of PD motor tasks [41,48]. Facial expression analysis has enabled quantification of hypomimia from short recordings [12], while speech tasks such as pangram utterances or sustained phonation capture dysarthria and articulation deficits [14,49]. Beyond PD, video has been applied to tremor quantification [50], gait analysis in ataxia [51], and chorea detection in Huntington disease [52]. Systems for facial asymmetry grading in Bell palsy highlight the broader clinical relevance of video-based pipelines [53]. Together, these studies demonstrate the feasibility of extracting clinically meaningful audio-visual features outside the clinic.

##### At-Home Assessments

Structured audio and video recordings of simple tasks, such as smiling, finger-tapping, or a pangram utterance (speech), are increasingly used for at-home PD assessments [21,23,54]. Similar tasks (hand movement, speech, and noise-to-finger test) are also applied in home-based ataxia assessment [18]. Models may analyze symptoms from a single task [29] or combine multiple tasks [17]. However, not all clinically informative tasks are practical for unsupervised use due to logistics and safety. For instance, the 10-meter walk test is standard for clinical assessment of PD [55] and ataxia [56]. However, this task requires ample space in front of the camera and poses fall risks for older adults [57,58]. By contrast, the finger-tapping, facial expression, and speech tasks are safe and simple for home deployment while probing core functional domains of neurological assessment, supporting continuous and accessible monitoring. However, unsupervised data collection introduces risks such as poor recording quality, incomplete execution, or noncompliance, which may degrade performance and compromise safety.

#### Quality, Compliance, and Safety in Multimedia-Based Screening

##### Impact of Recording Quality on Modeling

Model performance in A/V-based assessment is tightly coupled to recording quality. In computer vision, reductions in resolution, frame rate, signal-to-noise ratio, and blurring substantially reduce recognition accuracy [11]. Speech tasks are equally vulnerable to background noise and microphone artifacts. Improvements in low-resolution activity recognition with super-resolution [59], and correlations between deep quality metrics and streaming performance [60], confirm this dependency. Clinical studies similarly show that remote PD and ataxia ratings are reliable only when recordings meet minimum clarity and framing thresholds [18,41].

##### Device- and Environment-Induced Variability

Consumer devices and home environments introduce variability: cameras differ in resolution and frame rate, microphones in sensitivity and noise handling, and settings in lighting or background conditions [61]. Prior works show that many failures arise not only from hardware but also from usability barriers, such as unclear task instructions or difficulties in device positioning [62]. These issues are particularly acute for older adults, who face well-documented barriers in digital health adoption [63,64]. Addressing these challenges requires not only algorithmic solutions but also human-centered design to guide setup, reduce errors, and support compliance.

##### Safety and Trustworthy AI Safeguards

In real-world deployment, systems must abstain when quality is inadequate. Unlike controlled research datasets, poor-quality recordings can lead to unsafe misclassification and inappropriate reassurance or alarm. Advances in trustworthy AI emphasize calibrated confidence and abstention (ie, withholding or rejecting a downstream prediction when the input quality or model confidence is insufficient) [65], enabling systems to reject uncertain cases until higher-quality data are available. At the same time, human-computer interaction research emphasizes communicating these safeguards with transparency to preserve user trust [66,67].

#### Toward Automated Multimedia Quality Analysis in Movement Disorders

While existing automated multimedia (A/V) quality assessment methods focus on general visual or auditory properties (eg, blur, contrast, compression, or background noise) [68,69], these are insufficient for clinical use. Task execution and compliance, such as whether a smile is performed, a hand stays in frame, or speech is audible, are equally essential but rarely addressed. In medical contexts, the distinction between visual fidelity and diagnostic validity is increasingly recognized. However, few frameworks operationalize task-based metrics for video quality assessment in clinical screening and diagnosis [26]. Defining task-aware failure modes is nontrivial: systems must anticipate how recordings can go wrong (eg, hands leaving the frame, partial smiles, or missing speech segments) and detect these issues automatically [70]. Device heterogeneity and uncontrolled home environments further complicate consistent assessment. Our work addresses these gaps by extensively exploring three widely used movement-disorder tasks and delivering a holistic, task-aware quality assurance framework: (1) expert-curated guidelines derived from manual review of hundreds of unsupervised recordings, (2) automated, interpretable features explicitly tied to those guidelines, and (3) trained models for task-specific quality classification. We position automated task-aware quality analysis as a necessary safeguard. By filtering or abstaining from compromised inputs, systems can improve the reliability of video-based screening while reducing the risks of unsafe misclassification.

## Methods

### Participant Recruitment and Data Collection

In this study, we used data collected via PARK [71], a web-based platform designed for large-scale remote assessment of PD. The PARK platform allowed participants to enroll and complete study procedures outside of a clinic, primarily from their homes. Recruitment was conducted (from 2017 to 2025) through several predefined channels, including invitations sent through the University of Rochester Brain Health Registry, referrals from clinicians and research collaborators, and public outreach through social media and related online postings. These channels were used to reach both individuals with PD and individuals without PD. Eligible participants were required to be at least 18 years old, able to provide informed consent, and have access to an internet-connected laptop. Participants who self-reported having movement disorders other than PD were not enrolled. Sex and race/ethnicity were not used as eligibility or recruitment criteria. No formal sample-size calculation or enrollment ceiling was used. The study size was determined by the availability of willing and eligible participants who enrolled through the remote platform and completed the relevant recording tasks.

Interested participants accessed the PARK platform, reviewed the study information, completed an electronic informed-consent process, and provided demographic information and self-reported PD diagnosis status before completing the recording tasks. Because enrollment and participation occurred remotely, diagnosis status was not independently verified by a clinician as part of this study. The study did not include any follow-up data collection.

A total of 1444 confirmed eligible participants contributed demographic information and A/V recordings (primarily from home) across 21 standardized tasks spanning motor functions (eg, finger-tapping, hand movement, and fist making), facial expressions (eg, disgusted face, smile, and surprise), and speech (eg, sustained phonation, pangram utterance, and tongue twister). Before each task, the platform displayed an instructional video and task-specific on-screen prompts. Recordings were completed without real-time supervision, which allowed the study to capture the types of recording quality and task-compliance issues that can naturally occur in home-based multimedia assessment.

Among the participants, 750 identified as female, 609 as male, 1 as nonbinary, and 84 did not disclose their gender. The largest self-identified racial group was White (892/1444, 61.8%), followed by Black or African American (46/1444, 3.2%), Asian (45/1444, 3.1%), and smaller proportions identifying as American Indian or Alaska Native, Native Hawaiian or Pacific Islander, or multiracial (with over 20%, 289/1444) choosing not to disclose their race. The mean age of the participants was 61.7 (range 18-89; SD 13.7) years, reflecting the older demographic most affected by movement disorders. Among all the participants, 472 (32.7%) self-reported being diagnosed with PD (we do not have information regarding other movement disorders, as data were primarily collected for PD assessment).

### Ethical Considerations

The study was approved by the University of Rochester Research Subjects Review Board (RSRB/IRB) under protocol STUDY00001369 (Parkinson Remote Data). All participants provided informed consent for the use of their data in this research. Participants whose images are included in this manuscript also provided consent for the use of their images in research dissemination, including scholarly publications.

### Task Selection

We selected three standardized tasks for automated quality assurance:

1. Finger-tapping: Participants tapped their thumb against their index finger 10 times, performing the motion as quickly and as largely as possible. This classic assessment of bradykinesia [34,72] provides quantifiable indicators of motor speed and amplitude.
2. Facial expression/smile mimicry: Participants were instructed to produce a natural smile, return to a neutral expression, and repeat this sequence 3 times. This probes hypomimia [65,73], a reduction in facial expressivity observed across conditions such as PD, Huntington disease, and dystonia.
3. Speech/pangram utterance: Participants read aloud the sentences “The quick brown fox jumps over the lazy dog. The dog wakes up and follows the fox into the forest. But again, the quick brown fox jumps over the lazy dog.” containing a pangram encompassing all English letters. This task supports analysis of articulation, fluency, and clarity, which are frequently affected by PD, ALS, and other movement disorders.

We selected these 3 tasks based on their clinical relevance across movement disorders, feasibility for unsupervised home settings, and participant safety. Combined, these tasks capture complementary motor and speech modalities and are frequently used in clinical assessments of movement disorders. Finger-tapping is a core test for quantifying bradykinesia in PD [21]. It is also used to assess coordination deficits in Huntington disease [28] and cerebellar ataxia [29]. Facial expression tasks, such as smiling, capture hypomimia in PD [12], facial weakness in ALS [30], and dystonic movements in tardive dyskinesia [31]. Speech tasks, including sustained phonation and short reading passages, are routinely applied to measure dysarthria [74] and articulatory breakdowns [75] across PD, ALS, multiple system atrophy, and spinocerebellar ataxia. Tasks such as gait assessment [76], while well established in neurological research, pose practical challenges in remote contexts. Completing this task at home requires ample walking space in front of the camera and full-body capture, and can be unsafe (ie, risk of falling) for individuals with advanced mobility impairments. In contrast, the chosen tasks can be performed while seated, require only a webcam and microphone, and pose minimal safety concerns. Importantly, each task taps into functional domains commonly affected across a range of movement disorders: finger-tapping probes motor speed and coordination, smile mimicry captures facial expressivity, and pangram utterance reflects articulatory precision and prosody. In addition, the combination of these 3 tasks has recently been shown to support accurate detection of PD [17], further validating their practical utility for neurological assessments.

### Quality Annotation, Reference Labels, and Datasets

We recruited 3 annotators with 2-5 years of relevant expertise to establish task-specific guidelines and generate reliable datasets for automated quality classification. Two were doctoral students specializing in video-based movement disorder assessment, and the third was an undergraduate researcher experienced in analyzing home-recorded videos for PD. All annotators were familiar with the MDS-UPDRS [20] criteria, the clinical standard for evaluating PD, which supported consistency and credibility of their quality ratings.

Annotation proceeded in multiple phases. First, each annotator independently rated 50 randomly selected videos per task into three classes: 0 (poor), 1 (borderline), and 2 (good). Disagreements were resolved through collaborative discussion, during which the annotators jointly finalized labels for all videos. Based on this process, the team developed draft annotation guidelines tailored to each task. In the second round, they applied the guidelines to a new set of videos (71 for the finger-tapping task; 50 for the smile and speech tasks), again rating independently and resolving disagreements collaboratively. Only minor refinements of the guidelines were made after this stage, and interrater agreement improved significantly during the second round of ratings, with quadratic weighted Cohen κ (QWK) increasing by at least 0.23 across tasks (*P*<.001). Because recording quality and task compliance do not have an objective biological ground truth, we treated expert consensus labels as the reference standard for model evaluation. For the held-out test sets, 3 annotators first rated each recording independently using the task-specific guidelines. Disagreements were then resolved through collaborative adjudication, resulting in a single consensus reference label for each recording. These labels should therefore be interpreted as expert-derived consensus labels rather than absolute ground truth.

The borderline class was used for recordings that were not clear failures but contained minor quality or task-compliance issues that could reduce confidence in automated analysis. For example, speech recordings were rated as borderline when the participant remained intelligible despite background noise, a brief pause, limited repetition, or minor word omissions. Smile recordings were rated as borderline when the face remained visible despite mild blurriness, partial eyeglass glare, extra smiles, or slightly suboptimal camera distance. Finger-tapping recordings were rated as borderline when the hand remained mostly visible despite suboptimal lighting, partial wrist visibility, minor tap-count deviations, or slightly distant camera framing.

For analysis and deployment, the 3 quality classes reflect different levels of usability. Good-quality recordings can be passed confidently to downstream PD assessment models. Borderline recordings may still be usable, but their downstream predictions should be interpreted with caution; depending on the application, they may either be accepted with a warning or trigger corrective feedback and rerecording, reflecting the tradeoff between data quality and user burden. Poor-quality recordings indicate substantial quality or compliance failures and would prompt the user to rerecord.

For developing the training datasets, each annotator rated approximately one-third of the remaining videos following the final guideline. Overall, this process resulted in task-specific annotation guidelines, a reliable consensus-labeled test set, and relatively larger training sets for model development. A summary of the datasets is provided in Table 1.

**Table 1:** Summary of the quality assessment datasets for the 3 tasks we studied.

Task/split		Data, n	Quality classes, n (%)				Disease characteristics, n (%)		
			Poor	Borderline	Good	PD^a^		Non-PD	Unknown
Finger-tapping									
	Train	800	236 (29.5)	148 (18.5)	416 (52.0)	314 (39.3)		443 (55.4)	43 (5.4)
	Test	121	47 (38.8)	19 (15.7)	55 (45.5)	60 (49.6)		56 (46.3)	5 (4.1)
	Total	921	283 (30.7)	167 (18.1)	471 (51.1)	374 (40.6)		499 (54.2)	48 (5.2)
Smile									
	Train	798	182 (22.8)	171 (21.4)	445 (55.8)	340 (42.6)		433 (54.3)	25 (3.1)
	Test	100	22 (22.0)	26 (26.0)	52 (52.0)	45 (45.0)		52 (52.0)	3 (3.0)
	Total	898	204 (22.7)	197 (21.9)	497 (55.4)	385 (42.9)		485 (54.0)	28 (3.2)
Speech									
	Train	597	57 (9.6)	54 (9.1)	486 (81.4)	162 (27.1)		412 (69.0)	23 (3.9)
	Test	100	11 (11.0)	13 (13.0)	76 (76.0)	34 (34.0)		63 (63.0)	3 (3.0)
	Total	697	68 (9.8)	67 (9.6)	562 (80.6)	196 (28.1)		475 (68.2)	26 (3.7)

^a^PD: Parkinson disease.

### Task-Specific Annotation Guidelines

In this section, we describe how each of the 3 tasks was recorded, summarize the quality issues identified by expert annotators, and outline the task-specific guidelines developed by the team.

#### Speech Task

**Figure 2:**
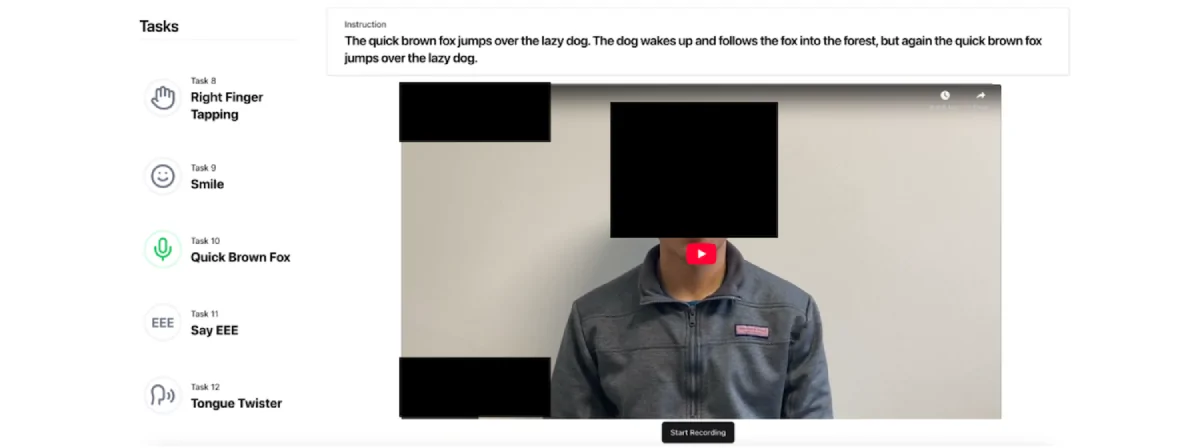
A screenshot of the PARK framework when collecting speech data. Each task data collection started with an instructional video. Participants could start recording themselves after watching the video.

For the speech task (pangram utterance), participants’ voices were recorded through their personal computer microphones using the PARK framework [71]. The framework first presents an instructional video explaining the task. During recording, the pangram sentence is shown on screen so participants can read it aloud rather than recite it from memory, a point emphasized in the instructional video. Figure 2 shows a screenshot of the recording interface.

Annotators reported several recurring issues affecting speech recordings. These included background noise or systematic noise caused by the microphone, low volume, unnatural pauses where participants appeared to forget the pangram and attempted to recite it from their memory (instead of reading from the screen), irrelevant filler words, and repeated words or phrases. In some cases, participants omitted several words or entire sentences. Finally, multiple voices were often captured in the same recording, primarily when another person assisted the participant.

Based on the review of 100 sample recordings and collaborative discussion among the annotators, a guideline (Table 2) was developed to rate the quality of speech recordings. Each recording was assumed to be of good quality (score=2) by default, with deductions applied for specific issues. A single-point deduction resulted in a borderline score (1), while a deduction of two or more points indicated poor quality (0).

**Table 2:** Speech task quality checklist.

Rule/issue			Penalty
Noise			
	Minor background/microphone noise only before task starts	OK	
	Noise during speech but intelligible	1 point	
	Noise interferes with understanding	2 points	
Pauses and memory lapses			
	One substantial pause suggesting recall; sentence otherwise mostly correct	1 point	
	Multiple pauses or insertion of irrelevant words	2 points	
Repetition			
	Limited repetition (3-5 words)	1 point	
	Excessive repetition or multiple complete attempts	2 points	
Omissions			
	Missing 3-4 consecutive words	1 point	
	Omission of a substantial portion (eg, entire clause or sentence)	2 points	
Multiple speakers			
	Two or more people speak simultaneously	2 points	

#### Smile Task

Similar to the speech task, participants first watched an instructional video with a demonstration, then recorded themselves using their computer webcam. During recording, an on-screen prompt instructed: “Make the biggest smile you can, followed by a neutral face. Slowly repeat this facial expression three times.”

**Figure 3:**
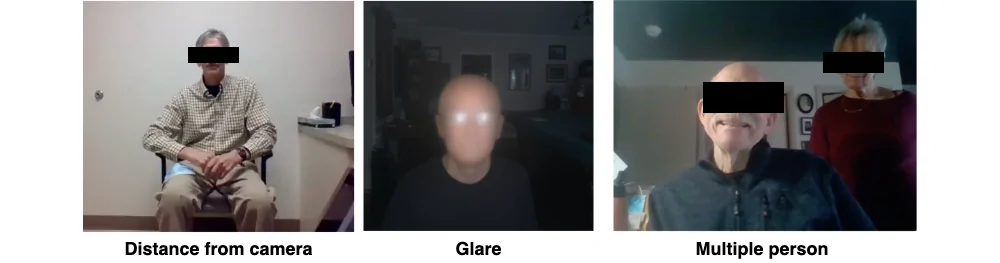
Quality issues in the smile task. Common problems included participants being too far from the camera, which limited the visibility of subtle facial movements for both human raters and AI-based pose tracking. Glare from eyeglasses often obscured the eyes, preventing reliable eye tracking and blink detection—important markers in movement disorder assessment. Another frequent issue was the presence of multiple people in the frame, which complicates automated analysis by requiring subject identification. The middle image represents real data but was generated by ChatGPT 5 [77] to preserve participant anonymity using the prompt “Generate an image of a 60-year-old male. The man is bald, wearing a black t-shirt, and sitting in a chair at his house. The background is a slightly dark living room (with windows, hanging photos, and other furniture). The man is wearing eyeglasses, and bright light is reflected from them. His eyes are severely obstructed due to these reflections.

Annotators noted several issues that compromised the quality of smile task recordings. Frequent problems included participants remaining idle for long periods, poor or uneven lighting, and multiple faces appearing in the frame. Glare from eyeglasses often obscured the eyes, sometimes severely limiting visibility. Other issues involved abrupt or overly fast smiles, excessive body movement that pushed the face out of frame, or participants sitting too far from the camera. In some cases, noncompliance was also observed, such as incorrect camera angles or failure to attempt the task. Representative examples are shown in Figure 3. Following a similar procedure as described for the speech task, a guideline (Table 3) was developed to rate the quality of smile videos.

**Table 3:** Smile task quality checklist.

Rule/issue			Penalty
Idle duration			
	Idle >5 s before or after the task	1 point	
	Idle ≥5 s between smiles	2 points	
Background contrast and blurriness			
	Dark/low-contrast background or blurry video, but face remains clearly visible	1 point	
	Face visibility impaired (unclear face)	2 points	
Multiple people in frame			
	Photos/pictures of other people visible in background	1 point	
	Another person’s face appears in the recording frame	2 points	
Eyeglasses			
	No glare or negligible glare	OK	
	Glare present, eyes remain partially visible	1 point	
	Glare severely obstructs visibility of either eye	2 points	
Smile count			
	Exactly 3 smiles	OK	
	4 or 5 smiles	1 point	
	<3 or >5 smiles	2 points	
Expression ambiguity			
	Smile resembles laughter or executed too quickly	1 point	
Body movement			
	Multiple head/body movements during smiles, but head always in the frame	1 point	
	Head leaves the frame during the task	2 points	
Distance from camera			
	Head-and-shoulders (headshot) framing	OK	
	Waist visible in frame	1 point	
	Lower body (eg, knees) visible; too far from camera	2 points	
Noncompliance (any of the following)			
	No smile-task recording; recorded on phone/tablet (instructed to use computer/laptop); face not fully visible; unstable/shaky camera; not forward-facing (bad angle); participant speaks during task	2 points	

#### Finger-Tapping Task

The finger-tapping task consisted of 2 separate recordings, 1 for each hand. After watching an instructional video with a demonstration, participants initiated their own recordings. During recording, the following instruction was displayed on screen: “Tap your index finger and thumb together on your [RIGHT/LEFT] hand 10 times as fast and as big as possible.”

**Figure 4:**
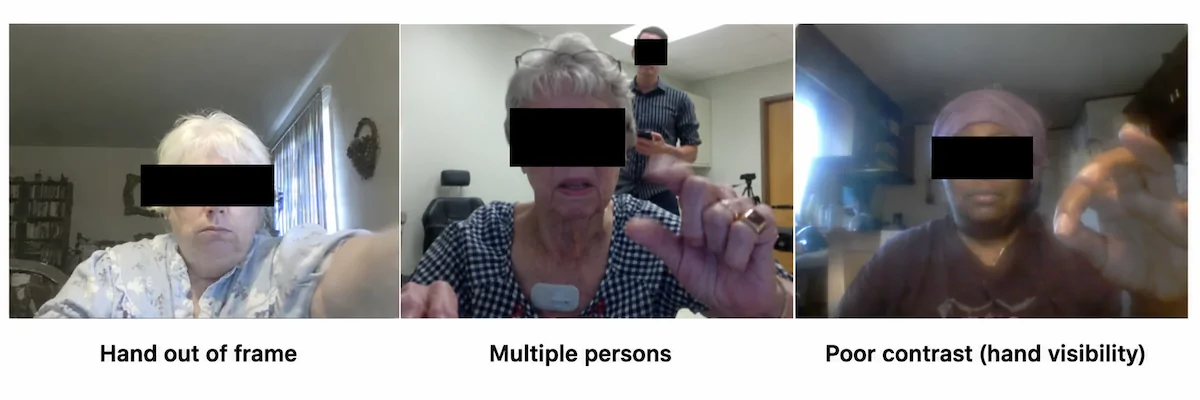
Quality issues in the finger-tapping task. Participants’ hands often moved out of the recording frame while tapping, and in some cases, multiple persons were visible, making it difficult for machine learning–based models to focus on the subject of interest. In addition, poor background lighting or contrast reduced hand visibility, which is crucial for reliable assessment of this task.

Annotators identified visibility as the most frequent challenge: some videos were blurry, poorly lit, or recorded with the participant too far from the camera, showing the torso or legs rather than focusing on the hand. In several cases, the wrist or hand moved out of frame, making taps difficult to assess. Tap count errors were also observed, ranging from minor (1-2 more or fewer than prescribed) to major deviations. Other issues included multiple hands appearing in the frame and instances of noncompliance, such as shaky cameras, idle behavior, or failure to perform the task (Figure 4). Based on these observations, the annotator team developed the guidelines (Table 4) for assessing the quality of finger-tapping recordings.

**Table 4:** Finger-tapping task quality checklist.

Rule/issue		Penalty
Background contrast and blurriness		
	Dark/low-contrast background or blurry video, but hand remains clearly visible	1 point
	Hand visibility impaired	2 points
Multiple hands in frame		
	Another hand visible in background but not tapping	1 point
	Background person also tapping	2 points
Distance from camera		
	Head-and-shoulders framing (headshot)	OK
	Waist visible in frame	1 point
	Lower body (eg, knees) visible; participant too far from camera	2 points
Wrist visibility		
	Wrist is briefly out of frame only at start/end; at least 5 consecutive taps fully visible	OK
	Wrist partially visible (not fully in frame)	1 point
	Wrist completely out of frame for >2 s during task	2 points
Tap count		
	8-12 taps (within ±2 of prescribed 10)	OK
	6-7 or 13-14 taps (deviation of 3-4)	1 point
	<6 or >14 taps (larger deviation)	2 points
Idle time		
	Idle period >5 s before or after the task	1 point
	Idle period >5 s during task execution	2 points
Noncompliance (any of the following)		
	No valid finger-tapping recording; invalid duration (too short ≤3 s or too long ≥20 s); aspect ratio inconsistent with standard computer webcams; unstable/shaky camera	2 points

### Automated Quality Assessment

#### NeuroSift Framework

In this section, we present the *NeuroSift* framework that automatically evaluates the quality and compliance of recordings across 3 standardized neurological tasks. The framework follows a multistage design. First, raw audio and video files are preprocessed for standardization. Next, task-specific interpretable features are extracted to reflect the expert-defined quality guidelines. Finally, machine learning models are trained on these features to generate ordinal, 3-class (ie, poor, borderline, and good) quality classification. This design ensures that automated predictions are both reliable and interpretable, while remaining aligned with the annotation criteria established by domain experts.

#### Preprocessing

Recordings were collected using the built-in microphones and webcams of participants’ devices. All raw recordings were initially stored in *webm* format. To ensure consistency across the dataset, video tasks were standardized to 15 frames per second (fps), resized to a resolution of 640×480 pixels, and re-encoded as mp4 files using the H.264 codec. For the speech task, *webm* files were converted to waveform audio format (*wav*) with a sampling frequency of 16 kHz. These preprocessing steps established a uniform input space for subsequent feature extraction and modeling.

#### Feature Extraction

We designed task-specific feature sets for speech, smile, and finger-tapping recordings to capture observable aspects of recording quality and task compliance. This design ensures interpretability and alignment with established human scoring criteria. The key feature categories for each task are summarized in Table 5. Full definitions are provided in Multimedia Appendix 1. The Python code for extracting these features is also publicly available (see the Data Availability section).

**Table 5:** Summary of task-specific features for automated quality assessment.

Task	Feature categories	Rationale
Speech	Speaker composition (multispeaker detection) Linguistic adherence (similarity, unique words) Temporal structure (audio and silence durations) Acoustic clarity (signal-to-noise ratio)	Capture deviations from the target sentence, missing or noisy speech, and multispeaker recordings that compromise intelligibility.
Smile	Face visibility (faces per frame, detection rate) Camera framing (waist/knee visibility) Lighting and reflection (eye detection, eyeglass ratio, brightness metrics) Task timing (smile onset/offset, idle time, smile count) Device context (aspect ratio)	Identify recordings with missing or multiple faces, poor illumination or eyeglass reflections, improper distance from the camera, and deviations from the 3-smile protocol.
Finger-tapping	Hand visibility (multihand ratio, wrist missing ratio, idle time) Movement compliance (tap count, duration, speed) Lighting (frame/hand brightness ratio) Camera framing (waist/knee visibility) Recording metadata (video duration, aspect ratio)	Detect recordings with missing or multiple hands, incorrect tap counts or speed, poor lighting, excessive distance from the camera, or nonstandard recording setups.

#### Quality Classification Models

We trained machine learning models to predict recording quality using 3 ordinal labels: poor, borderline, and good. As base classifiers, we evaluated adaptive boosting (AdaBoost) [78], light gradient boosting machine (LightGBM) [79], random forest [80], support vector machine (SVM) [81], and extreme gradient boosting (XGBoost) [82]. Because the borderline class contained relatively few samples, we also trained binary classifiers by merging the borderline with either the poor or the good class. To reflect different application needs, we defined 2 binary setups: a *restrictive* setting, where borderline recordings were grouped with poor quality, and a *flexible* setting, where borderline recordings were grouped with good quality—mirroring stricter clinical standards versus more lenient usability contexts. Recordings with missing task-specific features were excluded from model development and evaluation. This included 23 finger-tapping recordings, 16 smile recordings, and 28 speech recordings. On manual inspection, these recordings were either incorrectly formatted or did not contain the relevant task content, making reliable quality assessment infeasible.

The training pipeline included 2 preprocessing steps: feature selection and feature normalization. We ranked features using the *BoostRFE* algorithm from the *shap-hypetune* package and retained the top-K features (entirely based on the training dataset), followed by normalization with a *Standard Scaler*.

For the 3-class classification setup, sample-weighted loss functions were applied to mitigate class imbalance. Hyperparameters were tuned separately for each model family. For tree-based models (AdaBoost, LightGBM, random forest, and XGBoost), we searched over learning rates (1 × 10^−5^ to 10), maximum tree depth (2 – number of total features), number of estimators (25 to 1000), and top-*K* (2 to 10). For SVM, we additionally tuned the kernel type (linear, polynomial, or radial basis function) and the regularization parameter *C*.

To prevent information leakage, all data separation was performed at the participant level before feature selection, normalization, hyperparameter tuning, and model evaluation. Training and test sets contained recordings from nonoverlapping participants. For each task, a held-out validation subset was created from the training data and used only for model selection and hyperparameter tuning. Feature selection was performed using the BoostRFE algorithm from the shap-hypetune package using only the training subset. The selected feature set was then applied to the validation and test sets. Similarly, feature normalization was performed using a StandardScaler fit only on the training subset and then applied to the validation and test sets. Hyperparameters were selected based on QWK on the validation set. The held-out test set was not used during feature selection, scaling, hyperparameter tuning, or model selection, and all final performance metrics were reported only on this unseen participant-level test set.

#### Evaluation Metrics

Considering the ordinal nature of the 3-class quality labels (poor, borderline, and good), we adopted metrics that account for ordered outcomes in both interrater agreement analysis and model performance evaluation. In this setting, larger errors (eg, misclassifying good as poor) are penalized more heavily than smaller errors (eg, misclassifying good as borderline). Below, we summarize the metrics used.

##### Quadratic Weighted Cohen κ

The primary metric, QWK, measures agreement between two raters (or model vs annotators) while weighting errors according to their ordinal distance.

##### Agreement Rate (Accuracy)

The pairwise agreement rate measures the average proportion of exact matches across all rater pairs, while the complete agreement rate reflects the proportion of cases where all 3 annotators assigned the same quality label. For model evaluation, accuracy is defined as the proportion of predictions that exactly match the reference labels, providing a strict but intuitive measure of correctness.

##### Intraclass Correlation Coefficient

The intraclass correlation coefficient (ICC) was used to assess the reliability of interrater agreement by capturing both consistency and absolute agreement among the 3 annotators’ ordinal ratings. Higher ICC values indicate stronger agreement. We computed ICC using the 2-way mixed effects model for single raters, that is, ICC (3, 1).

##### Ordinal Classification Accuracy

Ordinal classification accuracy (OCA) measures performance while accounting for the ordered nature of the labels, so errors between adjacent classes, such as poor versus borderline, are penalized less than larger errors, such as poor versus good. OCA was derived from the *normalized match distance* [83] metric, originally proposed for ordinal quantification. It is defined as



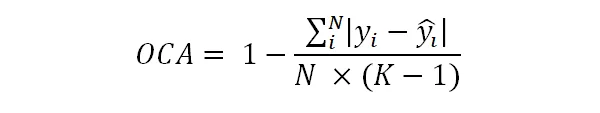



where *N* is the number of samples, *y_i_* is the ground-truth class label for the ith sample, 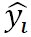 is the corresponding predicted class, and *K* is the number of ordinal categories. This metric is particularly well-suited for evaluating models in clinical and behavioral settings, where borderline cases are expected and minor disagreements are often acceptable.

For binary classification performance in the flexible and restrictive settings, we reported accuracy, precision, recall, *F*_1_-score, and the area under the receiver operating characteristic curve (AUROC).

#### Statistical Analyses

To evaluate the effect of the annotation guidelines on interrater reliability, we compared agreement metrics before and after guideline implementation. Agreement was assessed using QWK, pairwise agreement rate, complete agreement rate, and the ICC. Differences in agreement before and after guideline implementation were evaluated using the Mann-Whitney *U* test with bootstrap resampling.

For model evaluation, all performance metrics were computed on held-out test datasets consisting of participants not included in model training and validation. To account for variability induced by sampling and model randomness, we performed 1000 rounds of bootstrapping with replacement on the test set and reported the mean and SD for each metric.

For subgroup analyses, we compared model error rates across sex, age group, ethnicity, and self-reported PD diagnosis status. These subgroup analyses were conducted because demographic characteristics and disease status may influence recording quality, task performance, and model behavior in remote assessments. We used 2-proportion *z* tests when sample size assumptions were satisfied and Fisher exact tests otherwise. We also assessed the association between numeric age and per-sample prediction accuracy using Spearman correlation. Unless otherwise specified, all statistical significance tests were conducted at the 95% confidence level (α=.05).

## Results

### Reliability of Human Annotation

We first assessed interrater agreement on an initial set of 50 videos (per task), independently annotated by 3 experts without guidelines. Agreement metrics were then reassessed on a second set of 50+ videos (50 each for the smile and speech tasks; 71 for the finger-tapping task) after the introduction of structured, task-specific guidelines. As shown in Table 6, the guidelines significantly improved agreement across all tasks. In the finger-tapping task, QWK nearly doubled (0.46 to 0.89), pairwise agreement increased from 62.5% to 92.7%, and complete agreement rose from 47.8% to 89.9%. For the smile task, QWK improved from 0.61 to 0.84, and for speech, from 0.64 to 0.90. ICC values also increased consistently, with finger-tapping showing the largest jump (0.51 to 0.90). All improvements (Δ) were statistically significant (*P*<.001), based on the Mann-Whitney *U* test with bootstrap resampling. These results indicate that even experts initially struggled to annotate A/V data quality consistently, but structured guidelines provided a shared reference framework that reduced subjectivity, aligned judgments across raters, and enabled the creation of reliable training and test data for downstream modeling.

**Table 6:** Effect of guidelines on interrater reliability. Agreement metrics (mean and SD) before and after introducing structured guidelines for 3 tasks. Δ indicates the change. All improvements were statistically significant (*P*<.001).

Task/method		QWK^a^	Agreement rate (%)		ICC^b^ (95% CI)
			Pairwise	Complete	
Finger-tapping					
	Before guideline	0.46 (0.10)	62.5 (5.1)	47.8 (6.8)	0.51 (0.30-0.69)
	After guideline	0.89 (0.05)	92.7 (3.1)	89.9 (4.1)	0.90 (0.78-0.98)
	Δ	0.43 (0.10)	30.2 (5.1)	42.1 (6.9)	0.38
Smile					
	Before guideline	0.61 (0.08)	70.5 (4.9)	57.7 (7.0)	0.68 (0.53-0.80)
	After guideline	0.84 (0.04)	78.5 (4.4)	67.8 (6.6)	0.84 (0.76-0.91)
	Δ	0.23 (0.10)	8.1 (6.7)	10.1 (9.6)	0.16
Speech					
	Before guideline	0.64 (0.11)	81.9 (4.3)	73.9 (6.2)	0.65 (0.32-0.84)
	After guideline	0.90 (0.05)	91.9 (3.0)	87.9 (4.5)	0.91 (0.80-0.97)
	Δ	0.25 (0.12)	10.0 (5.4)	14.0 (7.9)	0.26

^a^QWK: quadratic weighted Cohen κ.

^b^ICC: intraclass correlation coefficient.

### Automated Quality Classification Performance

Building on the improved reliability of expert annotations, we next evaluated how well machine learning models could replicate these judgments on the 3-class quality prediction. Table 7 presents the performance of the best-performing model for each task (see Table S1 in Multimedia Appendix 2 for results from all 5 models tested; we also provide the confusion matrices for the best-performing models in Figures S1-S3 of Multimedia Appendix 3). For finger-tapping, *random forest* achieved the highest performance with QWK=0.71 and OCA=82.1%. For speech, *random forest* again performed best, reaching QWK=0.72 and OCA=89.9%. In contrast, smile proved more challenging: *LightGBM* was the top-performing model but with a comparatively lower QWK of 0.56 and OCA of 76.9%. Accuracy followed similar patterns, with finger-tapping and speech models showing higher consistency across classes compared to smile.

To better characterize model behavior under class imbalance, we further examined per-class performance for the 3-class models. Performance was highest for the poor and good classes and lower for the borderline class across all tasks. For speech, precision/recall/*F*_1_-score were 0.91/0.91/0.91 for poor, 0.38/0.38/0.38 for borderline, and 0.88/0.88/0.88 for good recordings. For smile, the corresponding values were 0.52/0.50/0.51 for poor, 0.30/0.23/0.26 for borderline, and 0.74/0.81/0.77 for good recordings. For finger-tapping, they were 0.82/0.77/0.79 for poor, 0.27/0.32/0.29 for borderline, and 0.78/0.78/0.78 for good recordings.

The confusion matrices in Multimedia Appendix 3 further show that most errors involved the borderline class. In the speech task, the dominant errors were borderline recordings classified as good and good recordings classified as borderline, while severe poor-versus-good confusions were rare. A similar pattern was observed for smile and finger-tapping, where many errors involved borderline recordings being assigned to an adjacent class or adjacent classes being assigned to borderline. This pattern suggests that the models generally captured the ordinal structure of the labels but struggled with intermediate-quality recordings, which are also the most ambiguous for human annotation and deployment decisions.

To support applications with different reliability requirements, we also trained models in 2 binary setups: a restrictive configuration (borderline merged with poor) and a flexible configuration (borderline merged with good). The restrictive setup is suited to contexts where ensuring data fidelity is critical. In contrast, the flexible setup allows for broader inclusion by treating borderline recordings as usable. Table 8 summarizes the results of the best-performing models for each task (refer to Table S1 in Multimedia Appendix 4, where we report the performance of all 5 models tested). In the restrictive setup, accuracies reached 81.8% for finger-tapping, 77% for smile, and 83.9% for speech, with AUROC values between 83.9 and 87.1. In the flexible setup, performance was generally higher, with accuracies of 83.5% for finger-tapping, 80.1% for smile, and 95% for speech. AUROC values were highest for speech at 99.5, demonstrating that the model was near-perfect in identifying poor speech recordings.

Overall, these results indicate that automated models can approximate expert ratings with substantial agreement, though performance varies across tasks and remains lower for the smile task.

**Table 7:** Performance (mean and SD) of task-specific quality classification models when evaluated on 3-class ordinal classification.

Task	Best model	QWK^a^	Accuracy	OCA^b^
Finger-tapping	Random forest	0.71 (0.06)	70.0 (4.1)	82.1 (2.7)
Smile	LightGBM	0.56 (0.07)	58.9 (4.9)	76.9 (2.9)
Speech	Random forest	0.72 (0.09)	81.8 (3.9)	89.9 (2.2)

^a^QWK: quadratic weighted Cohen κ.

^b^OCA: ordinal classification accuracy.

**Table 8:** Performance of task-specific quality classification models in binary setups. The metrics (mean and SD) represent their usual meaning (eg, precision, recall, and F1-scores are evaluated with respect to the good quality class). All values, including AUROC^a^, are reported on a 0-100 scale. Binary setup borderline→poor indicates that the samples from the borderline class were considered as poor quality during training and evaluation. Likewise, for the borderline→good setup, the borderline class was considered as good quality.

Task/binary setup			Best model		Accuracy		Precision		Recall		*F*_1_-score		AUROC
Finger-tapping													
	Borderline→poor	Random forest		81.8 (3.5)		78.7 (5.6)		81.8 (5.2)		80.1 (4.2)		87.1 (3.4)	
	Borderline→good	XGBoost^b^		83.5 (3.3)		83.7 (4.2)		90.7 (3.3)		87.0 (2.9)		86.6 (3.6)	
Smile													
	Borderline→poor	SVM^c^		77.0 (4.2)		73.2 (5.6)		88.2 (4.6)		80.0 (4.0)		83.9 (4.0)	
	Borderline→good	Random forest		80.1 (4.1)		88.3 (3.8)		85.9 (4.0)		87.0 (3.0)		77.3 (6.5)	
Speech													
	Borderline→poor	Random forest		83.9 (3.6)		90.5 (3.5)		88.1 (3.6)		89.2 (2.6)		84.4 (5.4)	
	Borderline→good	Random forest		95.0 (2.2)		100.0 (0.0)		94.3 (2.4)		97.1 (1.3)		99.5 (0.5)	

^a^AUROC: area under the receiver operating characteristic curve.

^b^XGBoost: extreme gradient boosting.

^c^SVM: support vector machine.

### Subgroup Analysis

#### Subgroup Definitions

To evaluate whether model performance varied across participant demographics and recording contexts, we conducted preliminary subgroup analyses for each task (smile, finger-tapping, speech) under the 3 above-mentioned classification settings. Subgroups were defined by sex (male vs female), age (<60 vs ≥60 years), ethnicity (White vs non-White), and PD diagnosis status (PD vs non-PD). In addition, we examined whether numeric age correlated with per-sample prediction accuracy using Spearman correlation. Because some subgroup sample sizes were small and some demographic groups were pooled, these analyses should be interpreted as preliminary evidence of subgroup performance rather than as definitive evidence of fairness.

#### Sex

Model performance was comparable between male and female participants across all tasks and classification settings. For the speech task, error rates were 15.9% (7/44) vs 22.8% (13/57) in the 3-class model, 9% (4/44) vs 1.8% (1/57) in the flexible binary, and 15.9% (7/44) vs 22.8% (13/57) in the restrictive binary settings. None of these differences were statistically significant (all *P*≥.16) according to *z* tests (of proportions) and Fisher exact tests (when sample size assumptions for normality were not satisfied). The finger-tapping and smile tasks likewise showed no significant sex-based performance difference (*P*≥.27 and *P*≥.48, respectively).

#### Ethnicity

Since individual non-White subgroups had limited representation, all non-White participants were grouped together. Even after pooling, sample sizes often did not meet normality assumptions, so Fisher exact tests were applied. In the 3-class model, a significant difference in error rates was observed for the speech task (*P*<.01), while finger-tapping and smile tasks showed no significant differences (all *P*≥.71). Under the flexible binary model, no ethnicity-related differences were detected across tasks (all *P*≥.22). In the restrictive binary model, speech (*P*<.001) and smile (*P*<.05) tasks showed significant difference in error rates, whereas finger-tapping task remained nonsignificant (*P*=.46).

#### Age

When participants were grouped into <60 years and ≥60 years, performance remained stable across tasks and models. Both *z* tests and Fisher exact tests indicated the absence of significant differences (all *P*≥.07). Spearman correlations between numeric age and per-sample accuracy were close to zero in the 3-class and flexible binary models for smile (Spearman ρ=0.03, −0.09; all *P*≥.38), finger-tapping (ρ=0.02, 0.09; all *P*≥.35), and speech (ρ=−0.15, 0.05; all *P*≥.13). In the restrictive binary finger-tapping model, a weak negative trend was observed (ρ=−0.18, *P*=.06), suggesting slightly lower accuracy with increasing age, though this was not statistically significant.

#### PD Diagnosis

We compared participants with and without a self-reported PD diagnosis to examine whether model performance was affected by the medical condition. Across all tasks and classification settings, no significant differences were observed between participants with and without PD, indicating that the models performed consistently within this dataset regardless of the diagnostic status of the participants providing the A/V samples (in all cases, *P*≥.11).

**Figure 5:**
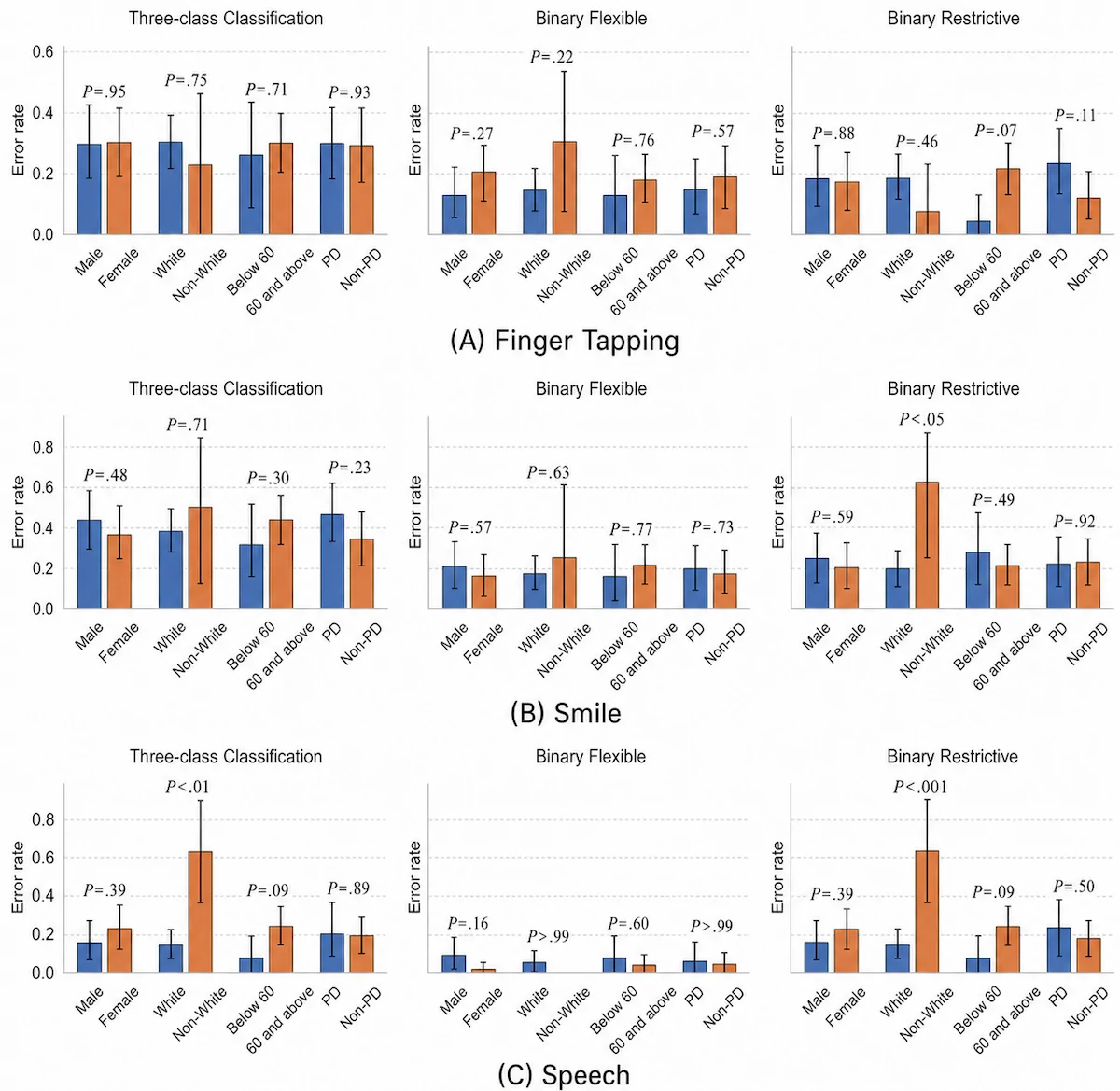
Group error rates across classification settings and task types. Each panel displays mean error with 95% bootstrap CIs for 2 subgroups (male vs female; White vs non-White; younger than 60 years vs 60 years and older; PD vs non-PD). Rows correspond to (A) finger-tapping, (B) smile, and (C) speech; columns to 3-class classification, binary flexible, and binary restrictive classifiers. *P* values are shown above each pair (significant when *P*<.05). Disparities are limited overall, with higher error for non-White users in speech (3-class and binary restrictive) and once in smile (binary restrictive); other comparisons are not significant. PD: Parkinson disease.

A complete overview of subgroup comparisons across modalities and classification settings is provided in Figure 5.

### Model Interpretability

**Figure 6:**
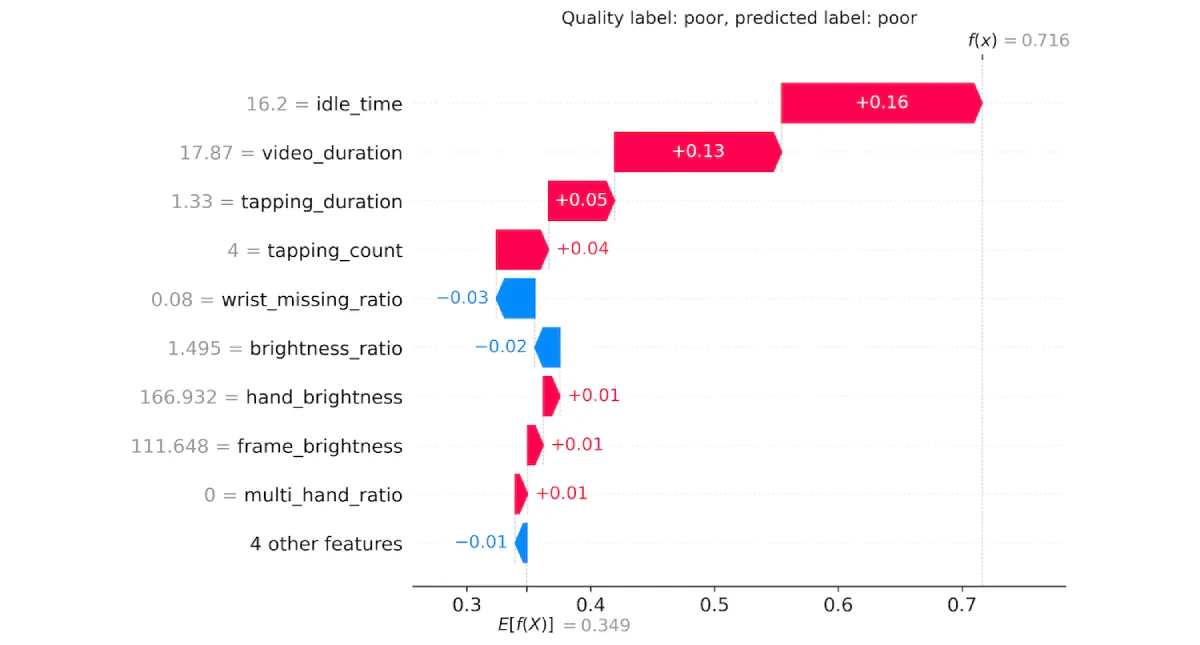
SHAP waterfall plot for a correctly classified finger-tapping recording. The model correctly predicted the recording as poor, with extended idle time, long video duration, short tapping duration, and a smaller number of taps (than prescribed) emerging as the strongest contributors. Other features, such as brightness ratio and wrist visibility, had minor effects. The explanation highlights specific, actionable issues that users could correct by rerecording (eg, reducing idle time and completing more taps). SHAP: Shapley additive explanations.

We used SHAP [32] to examine how individual features contributed to model predictions. Figure 6 shows an example in which the model correctly predicted a finger-tapping recording as poor. In this example, the largest drivers were extended idle time and unusually long video duration, while features such as brightness ratio and wrist visibility played smaller roles.

**Figure 7:**
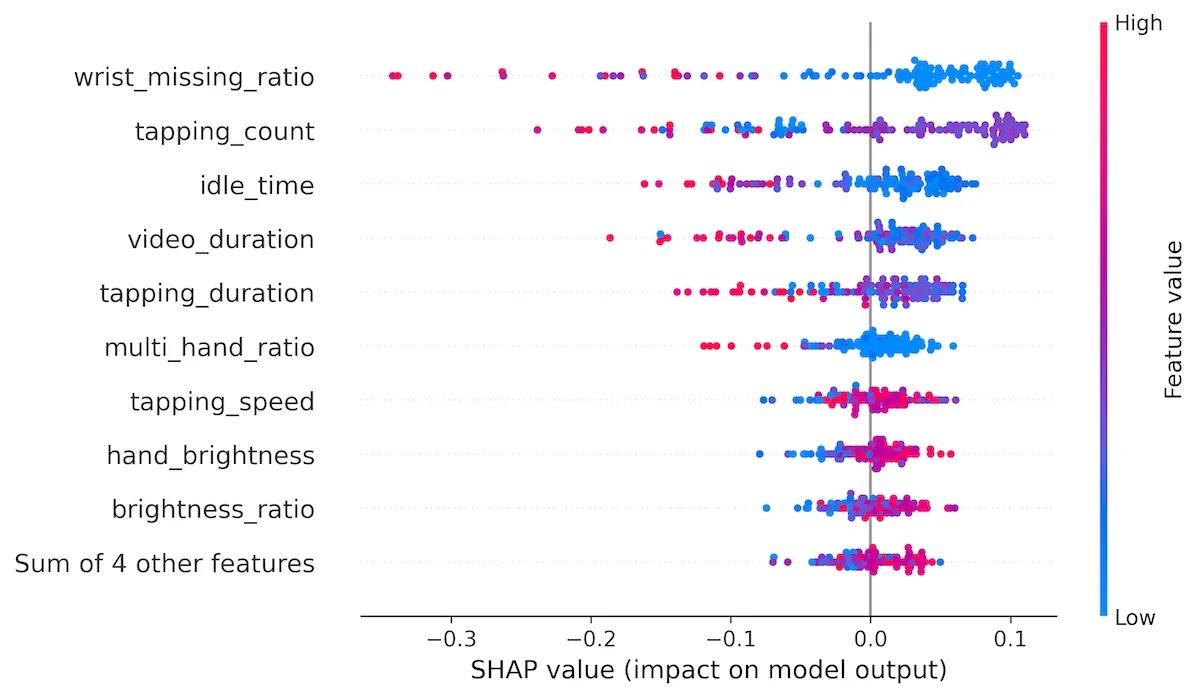
Global SHAP beeswarm plot for the finger-tapping task. Each point represents a sample, with color indicating the feature value (blue=low, red=high) and horizontal position showing the direction and magnitude of its impact. SHAP: Shapley additive explanations.

Beyond individual predictions, SHAP also highlighted global patterns in model behavior. As shown in Figure 7, features such as wrist visibility, tap count, and idle time consistently emerged as strong indicators of finger-tapping task quality. For the smile task, waist visibility and the ratio of eyeglass and face brightness played important roles (Figure S1 in Multimedia Appendix 5). For the speech task, similarity between the synthesized and target utterance, audio duration, and number of speakers were central contributors (Figure S2 in Multimedia Appendix 5).

## Discussion

### Summary of Main Findings

The aim of this study was to develop and evaluate NeuroSift as a task-aware, interpretable quality-assurance framework for user-recorded multimedia data in remote PD assessment. Specifically, we evaluated whether structured task-specific guidelines could improve expert agreement in quality annotation, whether interpretable features could support automated recording-quality classification across finger-tapping, facial expression, and speech tasks, and whether model explanations could identify actionable sources of recording failure. Overall, the findings support these aims. The annotation guidelines improved interrater agreement across all 3 tasks, suggesting that structured criteria can reduce subjectivity in quality assessment. The task-specific classifiers achieved moderate to strong performance against expert-derived consensus labels, with stronger agreement for finger-tapping and speech than for smile recordings. Finally, SHAP-based model explanations identified quality issues that were consistent with the expert guidelines, such as hand visibility, tap count, idle time, camera framing, eyeglass glare, and speech-related noise. Together, these findings suggest that task-aware quality assessment can provide a practical layer of quality control for remote multimedia-based PD assessment, while also identifying several areas that require further validation before clinical deployment.

### Interpretation and Implications

This study addresses an important challenge in remote digital health: unsupervised, user-recorded audio and video data can contain quality and task-compliance problems that are obvious to clinicians but difficult for automated systems to handle reliably. Unlike general video quality checks, NeuroSift focuses on task-aware safeguards. For example, it evaluates whether the hand remains visible during finger-tapping, whether facial expressions are adequately captured during the smile task, and whether speech is sufficiently audible and aligned with the target utterance. This distinction is important because a recording may be technically clear but still unusable for a specific clinical task if the relevant body part, movement, or speech segment is missing or poorly captured.

A central finding is that quality assessment itself can be subjective without structured criteria. The improvement in interrater agreement after guideline implementation suggests that explicit, task-specific annotation rules can provide a shared reference for raters and reduce ambiguity. This is particularly important for the borderline class, which represents recordings that are not clear failures but contain minor quality or compliance issues that may reduce confidence in downstream analysis. In practice, good-quality recordings can be passed more confidently to downstream PD assessment models, borderline recordings may be used with caution or may trigger corrective feedback depending on the quality-versus-user-burden tradeoff, and poor-quality recordings should prompt rerecording. This 3-level structure is more informative than a simple usable-versus-unusable decision, especially in remote settings where repeated rerecording may burden older adults or people with movement disorders.

The automated quality models reproduced expert-derived labels with varying performance across tasks. The stronger performance for finger-tapping and speech suggests that many quality issues in these tasks can be captured through interpretable features, such as wrist visibility, tap count, idle time, audio duration, speech similarity, and number of speakers. Smile quality assessment was more difficult, consistent with both lower postguideline interrater agreement and weaker model performance. This may reflect the inherent difficulty of evaluating facial expression quality in PD, where hypomimia can make it challenging to distinguish incomplete task performance from disease-related reduced expressivity. The per-class results and confusion matrices further suggest that borderline recordings are the most difficult to classify. Most errors involved borderline recordings being assigned to an adjacent class, or poor or good recordings being assigned to borderline, rather than severe poor-versus-good confusions. This pattern is consistent with the ordinal nature of the labels and supports the use of ordinal evaluation metrics.

Beyond PD assessment, the need for task-aware quality assurance is relevant to many user-recorded multimedia workflows. Remote education platforms often rely on live-streaming video presentations [84], digital recruitment systems request video responses to interview questions [85], and telehealth services routinely use patient-recorded videos for screening and monitoring [86,87]. In these settings, poor lighting, occlusion, background noise, or noncompliance with task instructions can affect both human interpretation and automated analysis. Prior work on telehealth usability and video-mediated communication also highlights that older adults and nonexpert users may face challenges in configuring devices, positioning themselves correctly, and producing usable recordings [88,89]. NeuroSift contributes to this broader design space by showing how quality guidelines, interpretable features, and feedback mechanisms can be aligned around task-specific requirements.

An important strength of NeuroSift is that its predictions can be traced to interpretable, task-relevant features. By surfacing the factors contributing to a quality prediction, the model can communicate not only that a recording failed but also why it failed. This information could be translated into actionable guidance, such as reducing idle time, completing more taps, improving hand visibility, or adjusting camera position.

However, raw SHAP plots are unlikely to be appropriate as direct feedback for participants, particularly older adults completing assessments without supervision. In deployment, these explanations would need to be translated into short, plain-language prompts linked to corrective actions. For example, features indicating poor hand visibility could be presented as “move your hand closer to the camera,” speech-noise features as “try recording again in a quieter room,” and face-framing or lighting features as “adjust the camera so your face is centered and clearly visible.” Locally deployed large language models could also be fine-tuned to translate SHAP-derived feature explanations into personalized, actionable feedback while avoiding transmission of raw audio or video data to external servers. Such feedback should avoid technical terminology, minimize cognitive burden, and limit repeated rerecording requests that may frustrate users. Future work should evaluate these feedback messages through user studies with older adults and people with movement disorders, assessing not only recording quality but also usability, trust, accessibility, and willingness to complete rerecordings.

These findings also have design implications for remote health tools. Quality assessment should not function only as a rejection mechanism; it can also become a user-support mechanism. Task-specific checks can be integrated into recording interfaces to provide real-time prompts before submission, such as asking users to recenter their hands, adjust lighting, or reduce background noise. Because NeuroSift uses lightweight models and interpretable features, quality screening may also be feasible directly on user devices. On-device assessment could reduce exposure of sensitive audio and video data while still providing immediate feedback. More broadly, by decomposing each task into measurable indicators of quality and compliance, task-aware safeguards could support remote assessment workflows in other health domains, including poststroke rehabilitation, speech or language therapy, and respiratory or mental health screening.

### Limitations

While our findings underscore the promise of automated quality assurance, several limitations remain. The dataset shows demographic imbalances, with limited representation of some racial and ethnic groups, which may affect model generalizability. Although the preliminary subgroup analyses suggested broadly equitable performance, the small number of minority samples reduced statistical power, and we observed significant discrepancies in speech quality classification across ethnic subgroups. These differences may reflect multiple factors, including dialectal variation, accent, microphone quality, background noise, and limitations of off-the-shelf automated speech recognition (ASR—a technology that converts spoken audio into text for analysis) modules [90], where racial disparities are well documented [91]. For example, African American participants may more frequently use African American Vernacular English [92], which is underrepresented in our dataset and could contribute to disproportionate outcomes if not carefully addressed. In deployment, quality assurance systems should avoid treating dialectal variation, accent, speech impairment, or differences in technology access as automatic evidence of poor-quality data. Low-confidence or borderline predictions may be better handled through corrective feedback, optional human review, adaptive thresholds, or alternative task pathways rather than automatic exclusion. Addressing these biases is critical, particularly because underrepresented groups and older adults often face greater barriers to accessing clinical care and may benefit substantially from home-based assessment. Future work should expand data collection among underrepresented groups, evaluate subgroup-specific error rates, calibration, and rejection rates, and develop strategies to mitigate bias in automated quality classification.

Residual uncertainty remained possible in the expert-derived reference labels, particularly for borderline recordings, where recordings may contain minor quality issues without being clearly unusable. This uncertainty is reflected in the postguideline agreement metrics, including complete agreement rates that remained below 90% across tasks. For the larger training datasets, each recording was labeled by a single annotator using the finalized guidelines, potentially introducing annotator-level label noise. We addressed this limitation by using structured annotation criteria, preserving the ordinal 3-class label structure, applying ordinal evaluation metrics, and evaluating final model performance on consensus-labeled held-out test sets.

Another limitation is the close coupling among the annotation guidelines, feature design, and model training. This coupling was intentional: the goal of NeuroSift was to produce interpretable quality predictions that could be traced back to observable recording-quality and task-compliance criteria. However, this design also means that the models primarily learn the expert-defined operational criteria encoded in the guidelines, rather than an independently established or universal definition of recording quality. Although we evaluated the models on held-out participants using consensus-derived reference labels, the reference labels and engineered features were still derived from the same annotation framework. Therefore, the present results establish internal validity within this quality-assessment protocol but do not fully establish external validity across different recording platforms, populations, environments, or annotation standards. Future work should validate NeuroSift on independently collected datasets and compare its predictions against alternative or independently defined quality criteria.

Although the predictive models aligned well with human raters for the finger-tapping and speech tasks, agreement was weaker for the smile task. While our feature sets were designed to closely mirror the quality assessment guidelines, they may not capture all relevant aspects of facial recordings. Some features (eg, detected peaks) depend on reliable smile detection, which is particularly challenging in individuals with PD due to hypomimia (ie, reduced facial expressiveness). Moreover, the smile task showed the lowest interrater agreement among the 3 tasks, even when raters followed the guidelines. Together, these findings suggest that smile quality assessment is inherently difficult, underscoring the need for richer feature representations and more robust annotation protocols for facial recordings.

NeuroSift generalizability requires further validation because all recordings were collected through the PARK framework. Importantly, however, PARK is a remote, web-based platform that captures recordings in participants’ natural home environments rather than under highly controlled laboratory conditions. Consequently, the dataset includes substantial variability in devices, browsers, lighting conditions, background noise, network environments, and user behavior, providing greater ecological validity than many laboratory-based datasets. Nevertheless, because all data were collected using a single platform and protocol, shared platform-specific characteristics may still influence feature distributions and model performance. Similar robustness concerns have been reported in movement disorder modeling, where small or heterogeneous datasets, class imbalance, and distributional artifacts can affect generalizability [93]. Although we used subject-independent test sets, consensus-derived labels, task-specific guidelines, and interpretable features, the present results establish internal validity within the PARK framework. Future work should evaluate NeuroSift on independently collected datasets and assess whether recalibration, domain adaptation, or platform-specific thresholds are needed to maintain performance across different recording environments. Beyond technical performance, the user experience of automated quality checks remains largely unexplored. Future work should examine both the benefits and burdens of rerecording requests through end-to-end studies that capture not only quantitative gains in data reliability but also participants’ qualitative perspectives on usability and trust.

Finally, this study did not empirically test whether NeuroSift improves downstream PD prediction performance, model calibration, or clinical decision-making. The present work focused on developing and validating the quality-assurance framework itself, rather than evaluating a complete diagnostic pipeline. A rigorous downstream analysis would require an independently evaluated PD assessment model, sufficient held-out data with complete task recordings and reliable clinical labels, and prespecified comparisons across quality-gating strategies. Future work should compare downstream model performance with no quality filtering, flexible filtering that retains borderline recordings, and restrictive filtering that accepts only good-quality recordings. Such studies should report not only predictive performance but also calibration, coverage, user rerecording burden, and impact on clinical workflow.

### Conclusions

In summary, NeuroSift demonstrates how task-aware quality assurance can be built into remote multimedia-based PD assessment. The broader implication of this work is that reliable remote health AI requires more than accurate downstream prediction models; it also requires safeguards that determine whether user-recorded data are sufficiently clear, complete, and compliant for analysis. By combining expert-derived quality guidelines, interpretable features, lightweight classifiers, and explanation-based feedback, NeuroSift provides a framework for making quality assessment more transparent and user-correctable. At the same time, the findings should be interpreted as validation of a quality-classification framework, not as evidence that downstream clinical prediction or deployment outcomes have already improved. Future work should test NeuroSift in independent datasets and real-world workflows, evaluate its effect on downstream prediction and calibration, and study whether its feedback mechanisms are usable and fair across diverse populations. If validated in these settings, task-aware and explainable quality assurance could become an important design layer for remote health systems that depend on user-generated multimedia data.

## Appendix

Multimedia Appendix 1 Task-specific features for automated quality assessment.

Multimedia Appendix 2 Three-class classification performance of all models.

Multimedia Appendix 3 Confusion matrices for the best models.

Multimedia Appendix 4 Binary classification performance of all models.

Multimedia Appendix 5 SHAP explanations for smile and speech tasks. SHAP: Shapley additive explanations.

Multimedia Appendix 6 STROBE checklist.

## Abbreviations

A/V: audio/video
AdaBoost: adaptive boosting
ALS: amyotrophic lateral sclerosis
ASR: automated speech recognition
AUROC: area under the receiver operating characteristic curve
ICC: intraclass correlation coefficient
IMU: inertial measurement unit
LightGBM: light gradient boosting machine
MDS-UPDRS: Movement Disorder Society–Sponsored Revision of the Unified Parkinson’s Disease Rating Scale
OCA: ordinal classification accuracy
PD: Parkinson disease
QWK: quadratic weighted Cohen κ
RF: radio frequency
SARA: Scale for the Assessment and Rating of Ataxia
SHAP: Shapley additive explanations
SVM: support vector machine
XGBoost: extreme gradient boosting

## Data Availability

The code and derived features supporting the findings of this study are available at GitHub [94]. The raw audio and video data are not publicly available due to privacy considerations. Supplementary materials are provided in the Multimedia Appendices.
