# Supplementary material for: NeuroSift for Task-Aware Quality Assurance of Multimedia Data in Remote Parkinson Disease Assessment: Machine Learning Model Development and Validation Study
Source: J Med Internet Res. 2026 Aug 18;28:e91756. doi: 10.2196/91756 (PMC13535310; doi:10.2196/91756)
Supplement: Supplementary file 2 [file jmir_v28i1e91756_app1.docx]

**Multimedia Appendix 1.** Task-specific features for automated quality assessment

##### Speech-Task Features

**(i) Number of speakers.** We used the pyannote/speaker-diarization model from Hugging Face [1] to identify the number of speakers.

**(ii) Similarity score.** We used the Whisper automatic speech recognition (ASR) model [2] to obtain transcriptions of the recorded speech and compared them against the reference pangram. The similarity score was computed with the SequenceMatcher function from Python’s difflib library, which measures normalized Levenshtein-based similarity [3].

**(iii) Unique word count.** We calculated the number of distinct words in each transcription (from Whisper) after removing punctuation and stopwords. Most participants produced nine unique words when the pangram was read correctly. In contrast, large deviations from this value indicated that the recording had missing or irrelevant words.

**(iv) Audio duration, and (v) silence duration.** Each recording typically consisted of an initial silence and an ending silence (which may be absent in some cases). To quantify how long participants actually spoke, we used the timestamps returned by the Whisper ASR model (millisecond-level precision) and segmented each recording into speech and non-speech regions. From this segmentation, we computed the total duration of the speech segment (audio duration) and the cumulative length of all non-speech regions (silence duration).

**(vi) Signal to noise ratio (SNR).** We used the initial one second from the first silent segment of the recorded audio (from start until the timestamp of the first word returned by Whisper) to estimate the background noise, and computed its power. We then applied a sliding-window approach (1-second window, 0.5-second step) to compute the average power of speech segments and compared it with the noise power. The SNR was calculated as

$SNR=10 {log}_{10} (\frac{P_{signal}}{P_{noise}})$

in decibels, where $P_{signal}$ and $P_{noise}$ denote the average power of speech and noise, respectively. In cases where no sufficient silence was available (e.g., the participant began speaking immediately), missing values were imputed using the 75th percentile of observed SNRs across the dataset.

##### Smile-Task Features

**(i) Mean faces detected, (ii) median faces detected, and (iii) face detection rate.** We applied MediaPipe face detection model [4] to each video frame, and extracted three features: the mean and median number of faces per frame, and the proportion of frames with at least one detected face. These measures captured common issues such as missing faces (e.g., participant sitting too far) or multiple faces (e.g., another person in the background). Both cases reduce the reliability of facial landmark extraction, making these features essential for verifying that the participant’s face was consistently captured.

**(iv) Waist and (v) knee visibility.** The smile task required a headshot view, with the face and upper shoulders clearly visible. When participants sat too far from the camera, their faces often appeared too small for reliable detection, and body parts such as the waist or knees entered the frame. To capture this issue, we applied MediaPipe Pose [5] and calculated the percentage of frames where waist landmarks (left and right hips) and knee landmarks (left and right knees) were detected with sufficient confidence. Frequent visibility of these landmarks indicated deviation from the headshot condition and reduced the reliability of facial landmark extraction.

**(vi) Eye detection rate, (vii) eyeglass ratio, (viii) face brightness, (ix) frame brightness, and (x) ratio of face and frame brightness.** Facial expression-based movement disorder assessment models often rely on fine-grained facial action units (AUs) [6,7]. Therefore, visibility of the face and eyes was critical. Recordings were often degraded by poor lighting (e.g., dim rooms, strong backlighting) or reflections on eyeglasses that obscured the eye region. To capture these issues, we extracted the following features using MediaPipe face and landmark detections: eye detection rate, the percentage of frames where eye landmarks were reliably detected; eyeglass ratio, defined as the mean grayscale intensity of the eye region divided by that of the face bounding box (unusually high values indicating eyeglass reflections); and face brightness, frame brightness, and their ratio, which together characterize illumination conditions, such as backlit settings (low ratio) or uniformly dark recordings (low brightness).

**(xi) Total duration, (xii) task duration, (xiii) idle time, and (xiv) ratio of task and idle duration.** We used OpenFace [8] to extract facial AUs and track the intensity of expressions throughout each recording. In particular, we focused on AU12 (lip corner puller), which is critical for detecting smiles. A smile was defined as at least three consecutive frames with AU12 activated (to reduce false positives from spurious detections). The first and last detected smiles defined the onset and offset of the task. From this sequence, we computed four temporal measures: the total recording duration, the task duration (time between first and last smile), the idle time (non-smiling segments), and the ratio of task and idle duration. These features quantified both the presence and the temporal extent of smiles.

**(xv) Detected peaks.** Although participants were instructed to smile three times, we observed deviations where more or fewer smiles were performed. To measure compliance, we analyzed AU12_r, the (continuous value) intensity of AU12. Threshold-based detection was unreliable due to inter-subject variance in AU12 intensity. We instead plotted the AU12 intensity curve and used GPT-4 [9] to estimate the number of clearly defined peaks.

The LLM was prompted with: “*Please analyze the curve in this image. Count the clearly defined peaks (where the curve rises from a low point to a high point and then falls back to a low point with clear intervals) and return only a single integer.*” Manual validation confirmed that this approach yielded reliable counts of major smile cycles, enabling us to assess adherence to the three-smile protocol.

**(xvi) Aspect ratio.** Although the smile task was intended to be recorded on a computer webcam, some participants used mobile phones or tablets, producing videos with different width-to-height ratios. To capture this, we computed the aspect ratio of each video. In cases where predictive movement disorder assessment models support multimedia data across different types of devices, the aspect ratio check may not be needed.

##### Finger-Tapping Task Features

**(i) Multi-hand ratio.** In the finger-tapping task, only one hand should be visible. The presence of multiple hands introduces ambiguity in landmark tracking and undermines the validity of tapping features. To capture this, we applied MediaPipe Hands [10] with a detection and tracking confidence threshold of 0.75 (set empirically). Then, we computed the proportion of frames in which multiple hands were detected.

**(ii) Wrist missing ratio and (iii) idle time.** The wrist is a critical landmark for finger-tapping analysis, since many derived features (e.g., tap angle formed by wrist, thumb tip, and index finger tip) depend on it. Because the task could be performed with either hand, we first isolated the target hand by running MediaPipe Hands across all frames and tallying detections with confidence greater than 0.9. The wrist missing ratio was defined as the proportion of frames where the target hand was detected but the wrist landmark was missing or out of bounds. Idle time was defined as the total duration (in seconds) with no detected hand. A high wrist missing ratio indicates unreliable wrist tracking even when a hand is visible. In contrast, long idle time reflects extended periods without a detected hand.

**(iv) Tapping count, (v) tapping duration, and (vi) tapping speed.** The finger-tapping task required participants to perform exactly ten taps. But in practice, some exceeded this number or tapped so quickly that taps were small and individual taps were difficult to distinguish. To quantify compliance, we computed a frame-wise angle sequence formed by the wrist, thumb tip, and index fingertip. The angle typically showed large, consistent oscillations for the finger-tapping task, with each transition from *>* 10^◦^ to _≤_ 10^◦^ corresponding to a tap (empirical observation). Based on this heuristic, we extracted three features: the total tap count, the elapsed time between the first and last tap (tapping duration), and the average tapping speed (taps per second).

**(vii) Frame brightness, (viii) hand brightness, and (ix) brightness ratio.** We computed the average grayscale intensity of the entire frame and of the detected hand region, and then derived their ratio. While landmark tracking was generally robust to brightness differences, this ratio helped identify cases where the hand appeared disproportionately bright or dark relative to the background.

**(x) Waist and (xi) knee visibility.** Using MediaPipe Pose, we calculated the proportion of frames in which waist or knee landmarks were detected, following the same approach as in the smile task. Frequent detection of these landmarks indicated that participants were seated too far from the camera, which reduced the visibility and reliability of hand movements during the tapping task.

**(xii) Video duration and (xiii) aspect ratio.** These features were included to detect recordings that were unusually long or short, as well as those captured on devices not complying with the study protocol. While these features do not affect hand landmark stability, they provide useful signals to flag recordings with atypical setups or device usage.

References

1. Jain SM. Hugging face. In Introduction to transformers for NLP: With the hugging face library and models to solve problems. Springer. 51–67 p.

2. Radford A, Kim JW, Xu T, Brockman G, McLeavey C, Sutskever I. Robust speech recognition via large-scale weak supervision. In: International conference on machine learning. PMLR. p. 28492–518.

3. Yujian L, Bo L. A normalized Levenshtein distance metric. IEEE Trans Pattern Anal Mach Intell. 29, 6:1091–5.

4. Kartynnik Y, Ablavatski A, Grishchenko I, Grundmann M. Real-Time Facial Surface Geometry from Monocular Video on Mobile GPUs. arXiv. 2019. doi: <https://arxiv.org/abs/1907.06724>

5. Bazarevsky V, Grishchenko I, Raveendran K, Zhu T, Zhang F, Grundmann M. BlazePose: On-device Real-Time Body Pose Tracking. arXiv. 2020. doi: <https://arxiv.org/abs/2006.10204>

6. Adnan T, Islam MS, Lee S, Chowdhury EMWR, Tithi SD, Noshin K, Islam MR, et al. AI-Enabled Parkinson’s Disease Screening Using Smile Videos. NEJM AI. 2025;2(7):2400950.

7. Ekman P, Friesen W V. Facial Action Coding System: A Technique for the Measurement of Facial Movement. Palo Alto (CA): Consulting Psychologists Press; 1978.

8. Baltrušaitis T, Robinson P, Morency LP. Openface: an open source facial behavior analysis toolkit. In: 2016 IEEE winter conference on applications of computer vision (WACV). IEEE; 2016. p. 1–10.

9. Achiam J, Adler S, Agarwal S, Ahmad L, Akkaya I, et al. GPT-4 Technical Report. arXiv. 2023. doi: <https://arxiv.org/abs/2303.08774>.

10. Zhang F, Bazarevsky V, Vakunov A, Tkachenka A, Sung G, Chang CL, et al. MediaPipe Hands: On-device real-time hand tracking. arXiv. 2020. doi: <https://arxiv.org/abs/2006.10214>.
