# Supplementary material for: NeuroSift for Task-Aware Quality Assurance of Multimedia Data in Remote Parkinson Disease Assessment: Machine Learning Model Development and Validation Study
Source: J Med Internet Res. 2026 Aug 18;28:e91756. doi: 10.2196/91756 (PMC13535310; doi:10.2196/91756)
Supplement: Supplementary file 3 [file jmir_v28i1e91756_app2.docx]

**Multimedia Appendix 2.** Three-class classification performance of all models

| **Task** | **Model** | **QWK** | **Accuracy** | **OCA** |
| --- | --- | --- | --- | --- |
| Finger-tapping | AdaBoost | 0.582 | 65.3 | 77.3 |
|  | LightGBM | 0.700 | 70.3 | 81.8 |
|  | Random Forest | 0.714 | 70.3 | 82.2 |
|  | SVM | 0.653 | 69.4 | 80.2 |
|  | XGBoost | 0.636 | 66.9 | 78.9 |
| Smile | AdaBoost | 0.524 | 58.0 | 75.5 |
|  | LightGBM | 0.571 | 59.0 | 77.0 |
|  | Random Forest | 0.541 | 58.0 | 76.0 |
|  | SVM | 0.506 | 62.0 | 76.0 |
|  | XGBoost | 0.408 | 54.0 | 72.0 |
| Speech | AdaBoost | 0.705 | 82.0 | 89.5 |
|  | LightGBM | 0.666 | 73.0 | 85.5 |
|  | Random Forest | 0.732 | 82.0 | 90.0 |
|  | SVM | 0.719 | 81.0 | 90.5 |
|  | XGBoost | 0.715 | 82.0 | 90.5 |

**Table S1.** Model ablations for three-class quality classification. Comparison of models across tasks using quadratic weighted Cohen’s kappa (QWK), standard accuracy, and ordinal classification accuracy (OCA). Best models (bold) are selected based on the QWK metric.
