# Supplementary material for: NeuroSift for Task-Aware Quality Assurance of Multimedia Data in Remote Parkinson Disease Assessment: Machine Learning Model Development and Validation Study
Source: J Med Internet Res. 2026 Aug 18;28:e91756. doi: 10.2196/91756 (PMC13535310; doi:10.2196/91756)
Supplement: Supplementary file 4 [file jmir_v28i1e91756_app3.docx]

**Multimedia Appendix 3.** Confusion matrices for the best models

(a) Three-class classification	(b) Binary classification (borderline →poor)	(c) Binary classification (borderline -> good)

**Figure S1.** Confusion matrices for the speech quality classification models.

(a) Three-class classification	(b) Binary classification (borderline →poor)	(c) Binary classification (borderline -> good)

**Figure S2.** Confusion matrices for the smile quality classification models.

(a) Three-class classification	(b) Binary classification (borderline →poor)	(c) Binary classification (borderline -> good)

**Figure S3.** Confusion matrices for the finger tapping quality classification models.
