# Supplementary material for: NeuroSift for Task-Aware Quality Assurance of Multimedia Data in Remote Parkinson Disease Assessment: Machine Learning Model Development and Validation Study
Source: J Med Internet Res. 2026 Aug 18;28:e91756. doi: 10.2196/91756 (PMC13535310; doi:10.2196/91756)
Supplement: Supplementary file 5 [file jmir_v28i1e91756_app4.docx]

**Multimedia Appendix 4.** Binary classification performance of all models

| **Task** | **Classification Approach** | **Model** | **Accuracy** | **Precision** | **Recall** | **𝐹1 score** | **AUROC** |
| --- | --- | --- | --- | --- | --- | --- | --- |
| Finger-tapping | Binary (borderline → poor) | AdaBoost  LightGBM  RandomForest  SVM  XGBoost | 75.21  79.34  81.82  79.34  78.51 | 67.61  75.86  78.94  72.73  75.44 | 87.27  80.0  81.82  87.27  78.18 | 76.19  77.88  80.36  79.34  76.79 | 76.21  79.39  81.82  80.0  78.48 |
|  | Binary (borderline → good) | AdaBoost  LightGBM  RandomForest | 85.95  80.99  84.3 | 88.0  81.48  84.81 | 89.19  89.19  90.54 | 88.59  85.16  87.58 | 85.02  78.64  82.5 |
|  |  | SVM | 78.51 | 77.27 | 91.89 | 83.95 | 74.67 |
|  |  | XGBoost | 83.47 | 83.75 | 90.54 | 91.46 | 87.01 |
| Smile | Binary (borderline → poor) | AdaBoost  LightGBM  RandomForest  SVM  XGBoost | 72.0  70.0  73.0  77.0  67.0 | 70.69  72.0  71.93  73.02  67.27 | 78.85  69.23  78.85  88.46  71.15 | 74.55  70.59  75.23  80.0  69.16 | 71.71  70.03  72.76  76.52  66.83 |
|  | Binary (borderline → good) | AdaBoost  LightGBM  RandomForest | 79.0  76.0  80.0 | 88.0  85.53  88.16 | 84.62  83.33  85.9 | 86.27  84.42  87.01 | 71.85  66.67  72.49 |
|  |  | SVM | 80.0 | 86.25 | 88.46 | 87.34 | 69.23 |
|  |  | XGBoost | 76.0 | 84.62 | 84.62 | 84.62 | 65.03 |
| Speech | Binary (borderline → poor) | AdaBoost  LightGBM  RandomForest  SVM  XGBoost | 76.0  76.0  84.0  81.0  79.0 | 88.24  88.24  90.54  85.19  86.67 | 78.95  78.95  88.16  90.79  85.53 | 83.33  83.33  89.33  87.90  86.09 | 72.81  72.81  79.50  70.39  71.93 |
|  | Binary (borderline → good) | AdaBoost  LightGBM  RandomForest | 91.0  92.0  95.0 | 98.78  100.0  100.0 | 91.01  91.01  94.38 | 94.74  95.29  97.11 | 90.96  95.51  97.19 |
|  |  | SVM | 91.0 | 97.62 | 92.13 | 94.80 | 86.98 |
|  |  | XGBoost | 94.0 | 97.70 | 95.51 | 96.60 | 88.66 |

**Table S1.** Model ablations for binary quality classification. The metrics represent their usual meaning (e.g., precision, recall, and F1 scores are evaluated with respect to the positive – good quality – class). Binary (borderline → poor) indicates that the samples from the borderline class were considered as poor quality during training and evaluation. Likewise, for Binary (borderline → good) approach, the borderline class was considered as good quality. For each model class, we choose hyper-parameters based on the AUROC score on the validation set. Best models (bold) are also selected based on the AUROC score.
