# Supplementary material for: NeuroSift for Task-Aware Quality Assurance of Multimedia Data in Remote Parkinson Disease Assessment: Machine Learning Model Development and Validation Study
Source: J Med Internet Res. 2026 Aug 18;28:e91756. doi: 10.2196/91756 (PMC13535310; doi:10.2196/91756)
Supplement: Supplementary file 6 [file jmir_v28i1e91756_app5.docx]

**Multimedia Appendix 5.** SHAP explanations for smile and speech tasks

(a) Importance of features for classifying the “good” quality videos

(b) A correctly classified instance

(c) Instance of an incorrect classification

**Figure S1.** SHAP explanation of automated quality classification of the smile task video.

(a) Importance of features for classifying the “good” quality audios

(b) A correctly classified instance

(c) Instance of an incorrect classification

**Figure S2.** SHAP explanation of automated quality classification of the speech task recording.
